# A Green Conformable Thermoformed Printed Circuit Board Sourced from Renewable Materials

**DOI:** 10.1021/acsaelm.3c00799

**Published:** 2023-09-18

**Authors:** Amirsoheil Honarbari, Pietro Cataldi, Arkadiusz Zych, Danila Merino, Niloofar Paknezhad, Luca Ceseracciu, Giovanni Perotto, Marco Crepaldi, Athanassia Athanassiou

**Affiliations:** † Smart Materials, Istituto Italiano di Tecnologia, Via Morego 30, Genova 16163, Italy; ‡ Dipartimento di Informatica, Bioingegneria, Robotica e Ingegneria dei Sistemi (DIBRIS), University of Genoa, Via all’Opera Pia 13, Genova 16145, Italy; § Department of Biology, University of Rome “Tor Vergata”, Via della Ricerca Scientifica, Rome 00133, Italy; ∥ Materials Characterization Facility, Istituto Italiano di Tecnologia, Genova 16163, Italy; ⊥ Electronic Design Laboratory, Istituto Italiano di Tecnologia, Via Enrico Melen, Genova 16152, Italy

**Keywords:** green electronics, cotton, poly(lactic acid), biopolymers, compostable materials, green manufacturing

## Abstract

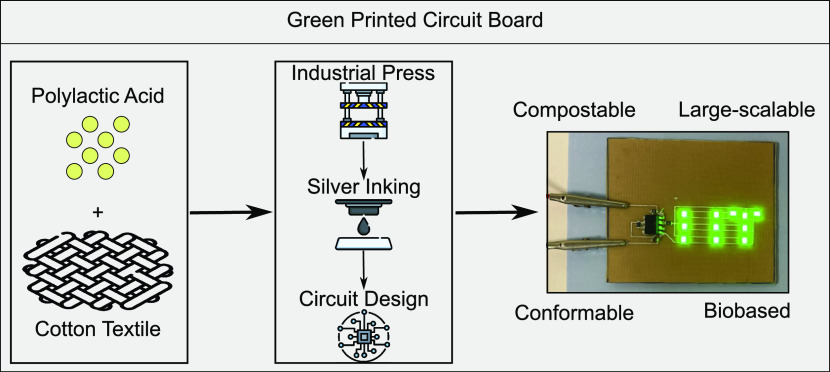

Printed circuit boards (PCBs) physically support and connect electronic components to the implementation of complex circuits. The most widespread insulating substrate that also acts as a mechanical support in PCBs is commercially known as FR4, and it is a glass-fiber-reinforced epoxy resin laminate. FR4 has exceptional dielectric, mechanical, and thermal properties. However, it was designed without considering sustainability and end-of-life aspects, heavily contributing to the accumulation of electronic waste in the environment. Thus, greener alternatives that can be reprocessed, reused, biodegraded, or composted at the end of their function are needed. This work presents the development and characterization of a PCB substrate based on poly(lactic acid) and cotton fabric, a compostable alternative to the conventional FR4. The substrate has been developed by compression molding, a process compatible with the polymer industry. We demonstrate that conductive silver ink can be additively printed on the substrate’s surface, as its morphology and wettability are similar to those of FR4. For example, the compostable PCB’s water contact angle is 72°, close to FR4’s contact angle of 64°. The developed substrate can be thermoformed to curved surfaces at low temperatures while preserving the conductivity of the silver tracks. The green substrate has a dielectric constant comparable to that of the standard FR4, showing a value of 5.6 and 4.6 at 10 and 100 kHz, respectively, which is close to the constant value of 4.6 of FR4. The substrate is suitable for microdrilling, a fundamental process for integrating electronic components to the PCB. We implemented a proof-of-principle circuit to control the blinking of LEDs on top of the PCB, comprising resistors, capacitors, LEDs, and a dual in-line package circuit timer. The developed PCB substrate represents a sustainable alternative to standard FR4 and could contribute to the reduction of the overwhelming load of electronic waste in landfills.

## 1 Introduction

PCBs are essential in the electronics sector because they physically support, connect, and interface electronic components and integrated circuits. PCBs represent the world’s 84th most traded product,^1^ and their market size surpassed USD 75 billion in 2021.^2^ The success of PCBs based on FR4 laminates derives from their excellent performance (i.e., dielectric, mechanical, and thermal) and their large-scale availability at a low price. Nevertheless, PCBs were designed in a linear economy context (produce, use, dispose) without considering sustainability and end-of-life aspects.^1^

In 2019, electronic waste (*e*-waste) production was approximately 7.3 kg per person per year excluding photovoltaic panels, and it is predicted to reach 74 Mt in 2030, being the fastest-growing class of waste.^3−5^ Improper handling of this kind of waste, such as through landfilling, can lead to the leaching and chemical spillage of toxic heavy metals such as tin and lead, posing a significant risk of soil and water contamination.^6,7^ Additionally, direct burning of *e*-waste results in the emission of toxic furans and dioxins, which have negative implications for both the environment and human well-being.^6,7^ PCBs waste makes up to 42% of the total *e*-waste weight, posing a significant environmental concern.^8,9^ Indeed, most PCBs are made of FR4, a laminate of glass fibers and epoxy resins. Such resins are thermosets, i.e., materials that cannot be recycled, and can contain brominated flame retardants, which are dangerous for the human neurological and reproductive systems and were found to be carcinogenic.^9−11^ Teflon, polyimide, polyester laminates, and ceramics are sometimes used as PCBs in niche application,^1^ but also in those cases the recycling is challenging because multiple materials are involved.^1^ As a result, only 20% of produced PCBs are recycled through costly and inefficient collection systems.^1^ In some cases, PCBs are burned in the landfills, generating debris or ashes that can enter groundwater, causing countless incidents of environmental damage.^3,12,13^ Considering all the above, the need for sustainable substrates for PCBs, compliant with the principle of green electronics^14^ and, thus, that can be safely disposed of or biodegraded at the end of life, is becoming paramount.^14−17^

Bio-based and/or biodegradable materials used as substrates for PCBs could preserve some of the advantages of FR4, such as light weight, robustness, easy fabrication, and low cost, while providing new opportunities such as flexibility, less energy requirement for their production, and significantly improved environmental impact.^14,18−22^ Deformability and conformability could be an advantage in some applications where complex geometries or mobile parts are needed, such as in robotics. Cellulose-based materials have been proposed so far in various works as green PCB substrates. Cellulose and nanocellulose substrates have many advantages, such as flexibility, biodegradability, recyclability, and low cost,^23−36^ but they suffer high roughness, moisture sensitivity, and poor barrier properties, all significant drawbacks in electronics, where smooth and humidity-insensitive substrates are required.^37^ Another approach to make green PCBs relies on including waste from the agricultural and farming sector such as lignin, rice husks, banana fibers, and chicken feather fibers, at high percentages reaching even more than 50% by weight, inside an epoxy resin.^38−42^ Nevertheless, epoxy resin is still present as binder in those substrates with all of the end-of-life issues previously described. Protein-based substrates such as silk or keratin based were also proposed as electronics substrates.^43−45^ Still, their extraction is commonly performed with multistep procedures at a lab scale, and such materials are vulnerable to water and other solvents. Recently, another material proposed for PCBs is fungal mycelium.^15^ Such substrates are natural, flexible, and biodegradable and were shown to be compatible with techniques, such as physical vapor deposition or laser patterning. Nevertheless, growing fungal mycelium with the desired roughness and physical features on a large scale still needs to be solved.

Large-scale produced biopolymers such as polycaprolactone, poly(ethylene glycol) (PEG), sodium alginate, cellulose acetate, or poly(lactic acid) (PLA) have also been used as substrates for degradable or transient electronics.^46−52^ In particular, thermoplastic polymers, such as PLA, are desirable for PCBs manufacturing because they are thermoformable and suitable for buildup by subsequent lamination of layers, a technique used in the development of conventional printed circuit boards, but can also be processed with additive manufacturing techniques for more advanced and niche applications.^53−55^ Nevertheless, so far such biopolymer substrates were fabricated mostly via multistep, not scalable solvent-based procedures, such as spin coating or chemical etching when integrated with electrical components.^46,51,55,56^ In particular, PLA substrates were often processed through toxic solvents such as chloroform.^57,58^ On the other hand, PEG or sodium alginate polymers are water-soluble and thus useful for transient applications, while they are not suitable for devices needing long-term ambient stability.

Considering the previous state of the art and the challenges derived from *e*-waste management, we present a green method for producing PCBs made from sustainable and compostable materials available on a large scale. The PCBs were designed by using only materials sourced from renewable resources. We used PLA biopolymer and cotton fabric to mimic the laminate structure of the traditional FR4. PLA is bio-based and compostable and currently has the largest production among the biopolymers, globally. Woven cotton fabric was selected because of its renewable origin and its large-scale production, compatible with the needs of the electronics sector. The PCB substrates were fabricated by melt-processing lamination without using solvents, taking advantage of the thermoplastic nature of PLA and the thermal stability of cotton fibers. The PCB substrates were subjected to various tests to examine their morphology, flexural and tensile strength, and electrical properties. We can anticipate that the electric properties of the substrate and the resistivity of silver tracks printed on top of it are comparable to those of standard FR4. The use of thermoplastic biopolymers enabled conformability to round surfaces by heating the substrates, preserving the conductivity of the conductive tracks to some extent. The proposed substrates could be a viable, greener, and conformable alternative to traditional PCBs.

## 2 Results and Discussion

**Figure 1:**
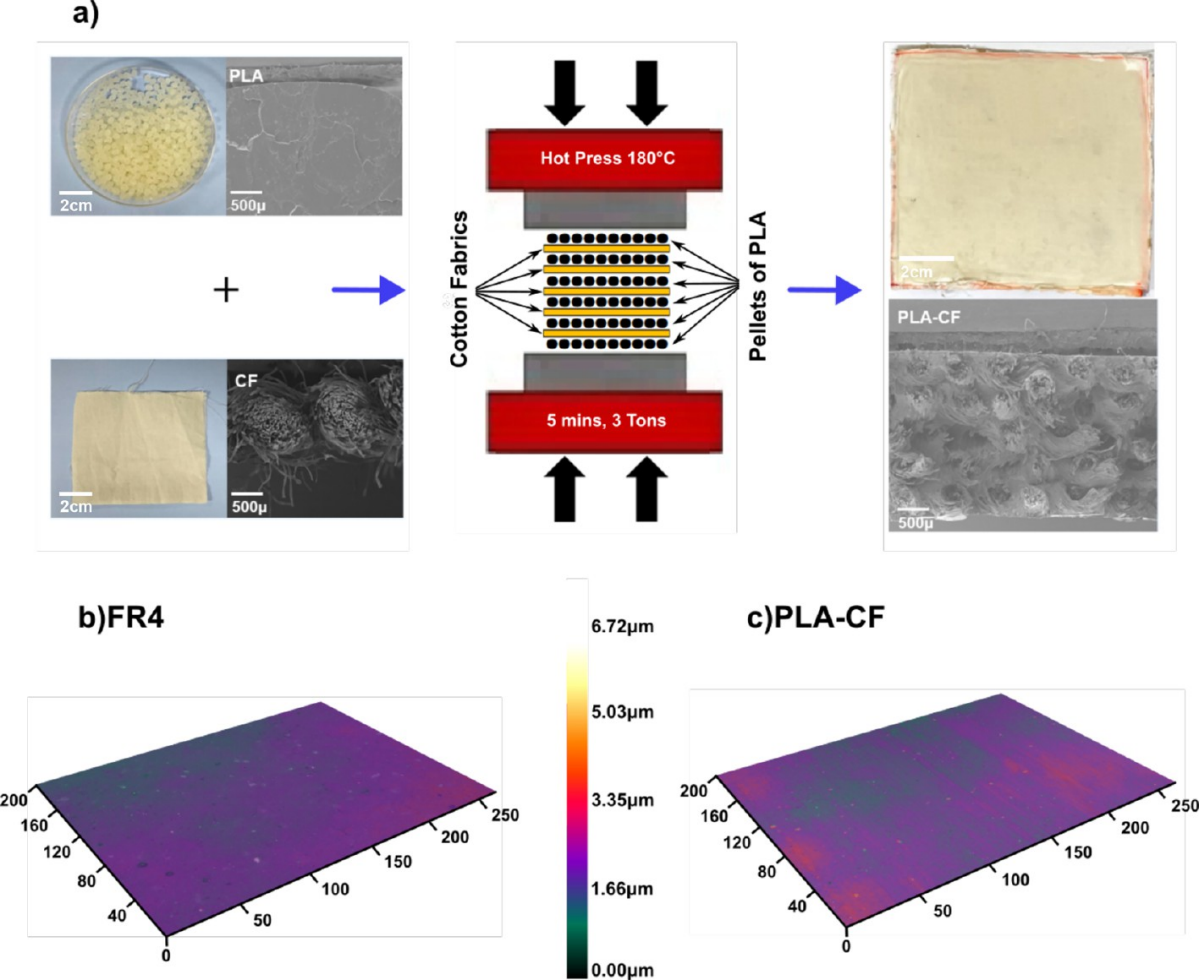
(a) Scheme of the preparation of the green PCB substrate. The bare PLA pellets and cotton fabric (CF) and the relative cross-section SEM are shown on the left. The manufacturing process is exhibited in the central plot. An image of the resulting bio-based PCB substrate (PLA-CF) and its cross-section is displayed on the right. (b, c) Height profile of the standard FR4 substrate and the PLA-CF green substrate, respectively. The *x*- and *y*-axes units are micrometers.

The PCB substrates were produced by lamination through compression molding (Figure 1a). Alternating layers of PLA pellets and cotton fabrics were inserted between the plates of the hot press. The arrangement of the layers was six layers of PLA pellets, two external and four internal, alternated by five layers of cotton fabric. The press temperature was set at 180 °C to melt the PLA and infuse it within the weave of the cotton fabric.^59^ As shown in the differential scanning calorimetry in Figure S1, the melting temperature of PLA is *T*_m_ ≈ 150 °C. This temperature is far below the temperature at which both cotton and PLA^60^ degrade, as confirmed by the TGA in Figure S2, which demonstrates that the weight loss starts at 300 °C. Such a feature is crucial for the integration of the circuit components to the PCB substrate, considering that many soldering alloys, including eutectic 63Sn-37Pb, melt below 300 °C.^61−63^ Low-temperature (138 °C) Sn42/Bi57.6/Ag0.4 tin–bismuth-based solders, used when low manufacturing temperatures are necessary, could be the best choice for our substrate. The cross section of the resulting material, shown at the bottom right of Figure 1a (sample labeled PLA-CF), demonstrates that the layers of the cotton fabric were fully bonded and interpenetrated by the layers of PLA without any defects or voids.

Surface roughness of the PCB substrate is an important parameter that determines the printing quality of the circuits using conductive inks or paints.^16^ For this reason, the surface morphology of the developed bio-based PCB substrate was compared with the one of standard FR4 through SEM and profilometer tests, shown in Figures S3 and 1b,c, respectively. The SEM revealed a flat and uniform surface for both substrates at the tens of micrometers length scale. Also, the profilometer images of the two substrates were very similar, while the surface roughness extracted from those images was 2.39 ± 1.27 μm for the FR4 substrate and 2.67 ± 1.52 μm for the PLA-CF substrate (see Figure S4). We can conclude that with the method used in this work, the roughness of the developed green composite material is equivalent to that of the conventional substrate, with the PLA-CF substrate having a broader value distribution compared to the FR4, as evident in the graphs. Note that the roughness of the PLA-CF substrate may be adjusted by using a smoother pressing surface with a hot press.

**Figure 2:**
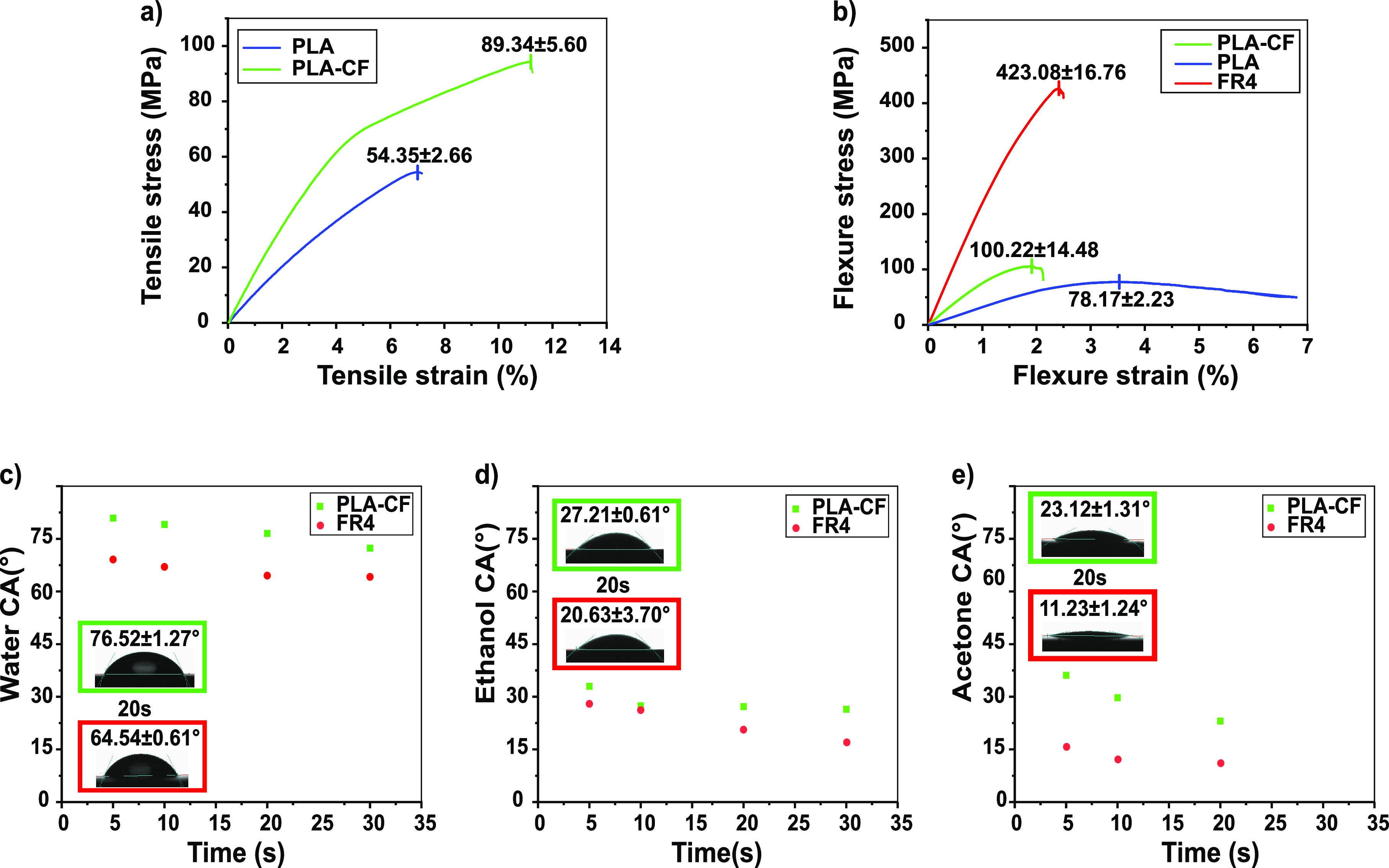
(a) Tensile stress–strain curves of pure PLA and of the PLA reinforced with cotton fabric (PLA-CF). (b) Flexural bending test of the pure PLA, FR4, and PLA-CF. (c–e) Water, ethanol, and acetone contact angles of PLA-CF and FR4 as a reference.

The mechanical properties of the developed green substrate were evaluated through tensile stress–strain (Figure 2a) and flexural (Figure 2b) tests and compared with those of pure PLA and the conventional FR4 substrate when possible.

In particular, the tensile stress–strain tests were performed on the developed green PCB substrate, pure PLA, and cotton fabric as a reference (Figure S5). The conventional FR4 substrate could not be tested with our gripping method because the instrument clamps could not hold the FR4 substrate and apply tensile force. The mechanical characteristics of pure PLA are typical of stiff thermoplastic polymers, with Young’s modulus 1.10 ± 0.04 GPa, elongation at break 6.72 ± 0.26%, and a tensile strength of 54.35 ± 2.66 MPa, as shown in Figure 2a and Table S1. Cotton fabric significantly reinforced the PLA-CF green composite, nearly doubling its strength (89.34 ± 5.60 MPa) and Young’s modulus (1.80 ± 0.23 GPa) and extending the elongation at break to 11.98 ± 1.17%.

The flexural tests were performed on the green PLA-CF PCB substrate, pure PLA, and FR4 as a reference, as shown in Figure 2b and Table S2. The pure PLA showed a flexural strength of 78.17 ± 2.23 MPa with a flexural modulus of 3.26 ± 0.17 GPa. The PLA-CF sample displayed a reinforcement, with a flexural strength of 100.22 ± 14.48 MPa and a modulus of 7.47 ± 1.09 GPa. On the other hand, the flexural strength of FR4 was found to be 423.08 ± 16.76) MPa, and the modulus is 22.66 ± 0.82 GPa. From the results presented above, we can conclude that the inclusion of cotton significantly boosted the mechanical properties of the PLA-CF substrate of the green PCB with respect to pure PLA from all the points of view (modulus, strength, and elongation at break), while the developed substrate is more flexible than FR4.

The wettability of the surface of PCBs is another important parameter to measure because it affects the printability of the conducting inks for the formation of the circuits. One of the characteristics that determines suitable wetting is the ability of the ink to spread to a satisfactory extent and to display adequate adhesion to the substrate, forming a continuous pattern.^64^ Good wetting is a prerequisite for crackless and homogeneous printed patterns.^64^ Contrarily, excessive spreading increases the patterns’ width and limits resolution.^64^ An indication of the surface wettability is given by the contact angle (CA) of a liquid drop onto the surface.^64,65^ There are four CA wettability regimes:^64−66^ (1) CA ≈ 0° → complete surface wetting with the liquid spreading onto the surface, resulting in unwanted smearing. (2) CA < 90° → the surface is liquid-philic with partial wetting and in electronics is the desired wetting regime. (3) CA > 90° → the surface is liquid-phobic, and the wetting is not optimal. (4) CA > 150° → the surface is super-liquid-phobic, and there is no wetting.

The contact angle was tested for three representative liquids that can be found in many inks used in electronics:^16^ water, ethanol, and acetone. Their CA data are presented in Figures 2c, 2d, and 2e, respectively, and in Table S3. The measurements were performed on PLA-CF samples and on FR4 for comparison. The water and ethanol contact angles were measured 5, 10, 20, and 30 s after the droplet contacted the surface, while for the acetone, it was measured up to 20 s due to fast evaporation. Figure 2c shows the water CA results, demonstrating that both the PLA-CF substrate and FR4 showed hydrophilic behavior. For example, as shown in the figure inset, at 20 s, the water CA was 76.52 ± 1.27° and 64.54 ± 0.61° for the PLA-CF and FR4, respectively. The ethanol and acetone contact angles were lower than the water ones on both substrates due to their lower surface tension (i.e., 72 mN/m for water, while ethanol and acetone have 22 and 23 mN/m, respectively).^67^ For example, at 20 s, the ethanol CA was 27.21 ± 5.34° and 20.61 ± 3.70° for the PLA-CF and FR4, respectively, while the acetone CA was 23.12 ± 1.31° and 11.23 ± 1.24° for the PLA-CF and FR4, as shown in the inset of Figures 2d and 2e, respectively. All the CAs appear to be relatively stable with time, an important feature that allows the ink to be stabilized on the substrate after its deposition. It is worth noting that all the CAs on the PLA-CF fall into the partial wetting liquid-philic regime, which generally leads to the best printing results.

**Figure 3:**
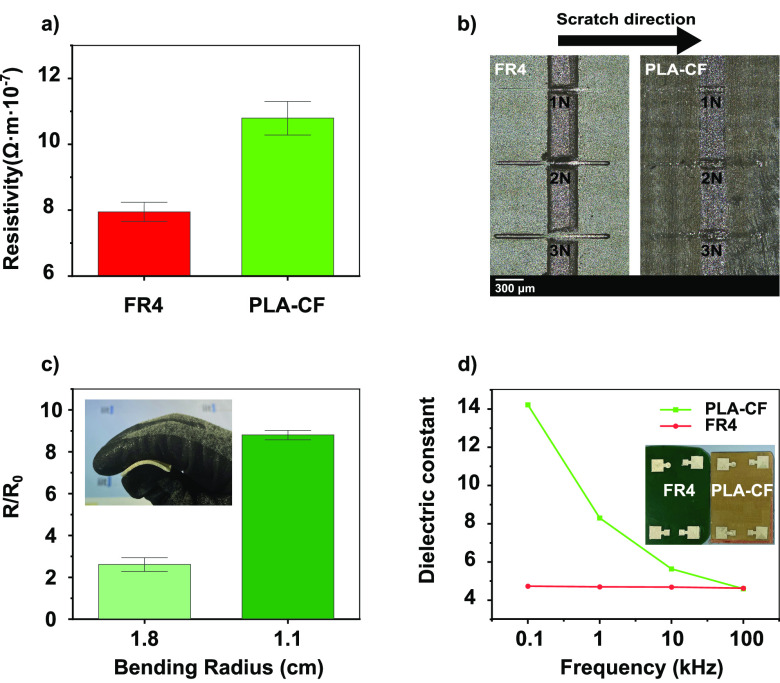
(a) Resistivity of printed silver lines on top of FR4 and poly(lactic acid) cotton fabric (PLA-CF) green PCB substrate. (b) Optical image of the scratched surface on the inked FR4 and PLA-CF substrate with forces ranging from 1 to 3 N. (c) Change in the resistance *R*/*R*_0_ of the printed silver lines on the PLA-CF substrate with bending. (d) Dielectric constant as a function of the frequency of the PLA-CF and FR4 substrates.

The continuous printing of electrically conductive inks is essential for effective electrical connections between electronic components on the PCB surface. To test the efficacy of such printing, we used commercial silver ink printed on the surface of PLA-CF and FR4 substrates, using the Voltera V-One Flexible Conductor 2 ink^68^ and comparing the results in terms of the morphology of the printed lines and of the electrical resistivity obtained. The results are shown in Figures S6 and 3a, respectively. The comparison was done using printed ink lines of 2.00 cm length and 0.36 mm width on each substrate. The morphologies of the conducting lines are similar, and there is no apparent smearing on any of the substrates. The thickness for both lines was about 0.04 mm, as shown in Figure S6. Silver particles appear slightly less evenly distributed on top of the PLA-CF sample, possibly due to the small difference in roughness between the two substrates, as evidenced in the profilometer analysis (see Figure 1). This effect can be minimized using a smoother surface in contact with the PLA-CF upon hot pressing. The *I*–*V* curves of the conducting lines showed an ohmic behavior (Figure S6), and the resistivity was calculated considering the geometry as explained in the Methods section (see eq 1). As shown in Figure 3a, the resistivity was (7.94 ± 0.78) × 10^–7^ and (10.80 ± 0.59) × 10^–7^ Ω·m for the FR4 and green PCB substrate, respectively. The difference is most likely due to the different roughness of the two substrates, but both values are in line with the state of the art of printed metallic conductive tracks on top of PCB substrates.^16^

Adhesion of the printed electrically conductive lines is another crucial feature for the stability of the circuits. Thus, we tested the durability of the drawn lines on top of the substrates against scratching, a stress that may occur in real life applications. The scratches were made by applying a constant load of 1, 2, and 3 N transversely to the drawn lines as shown in Figure 3b. The electrical resistance of the lines across the scratch point was tested before (*R*_0_) and after the scratch (*R*) (see Table S4). With 1 and 2 N scratch force applied, the green PCB and FR4 substrates showed *R*/*R*_0_ values of approximately 1. Conversely, applying 3N force, FR4 completely lost conductivity while the PLA-CF PCB showed a result similar to the one obtained with smaller loads. The loss of conductivity for FR4 is due to the removal of the printed line, indicating a lower adhesion of the ink to this substrate compared to the developed green PCB substrate. This behavior is most likely due to the annealing step performed at 60 °C (see the Methods section) to cure the silver ink. In the case of the developed green substrate, the used PLA biopolymer goes through its glass transition at ≈60 °C (see Figure S1), improving its interaction with the silver ink. On the other hand, the conventional FR4 substrate remains unaffected at that temperature.

Taking advantage of the glass transition of the biopolymer matrix of the developed green substrate, which is around 60 °C,^69^ a straightforward shaping of the green PCB can be enabled by increasing the temperature close to this value. Indeed, using a heat gun (see Video S1; the approximate temperature reached with the heat gun procedure is around 80 °C), we could heat-shape the green PCB conformally to diverse curved surfaces (i.e., bending radius ranging from 1.8 to 0.7 cm). On the other hand, with the same procedure the FR4 substrate was impossible to be reshaped because epoxy resins have a much higher *T*_g_, ranging from ≈130 to 180 °C.^70^ After reshaping the green substrate, the electrical resistance of the conductive silver tracks printed on it was measured, and the results are displayed in Figures 3c and S8. At the bending radii of 1.8 and 1.1 cm, the resistivity ratio of the conductive lines *R*/*R*_0_ becomes 2.61 ± 0.36 and 8.81 ± 0.20, respectively. Bending of the substrate at a radius of 0.7 cm led to a complete loss of the electrical conductivity due to significant crack formation and removal of the conductive silver from the substrate, as shown in Figure S9.

We evaluated the dielectric constant of the green PCB and compared it with the reference FR4. We printed plane capacitors of known geometries using silver ink on the surfaces of both substrates, shown in the inset of Figure 3d. We measured the capacity at different salient frequencies for applications (i.e., ranging from 0.1 to 100.0 kHz, the instrument’s limit) and calculated the dielectric constant using eq 2 in the Methods section. Increasing the frequency, the dielectric constant of the green PCB decreased from 14.22 ± 1.14 to 4.60 ± 0.14. In particular, we noticed that at the highest frequencies measured (i.e., 10 and 100 kHz), the dielectric constant is close to the value of the FR4 substrate. Indeed at 10 and 100 kHz, the dielectric constant of the PLA-CF was 5.6 and 4.6, respectively, while the value for the FR4 was constant at 4.6 in the entire range of frequencies measured, as shown in Figure 3d and Table S5. Notably, the decrease of the dielectric constant with increasing frequency occurs to all dielectric materials, above a specific frequency.^71^ This effect is due to the material’s net polarization drop, as dipole moment alignment cannot keep pace when the frequency becomes too high. This process is negligible with commercial FR4 at the used frequencies.

**Figure 4:**
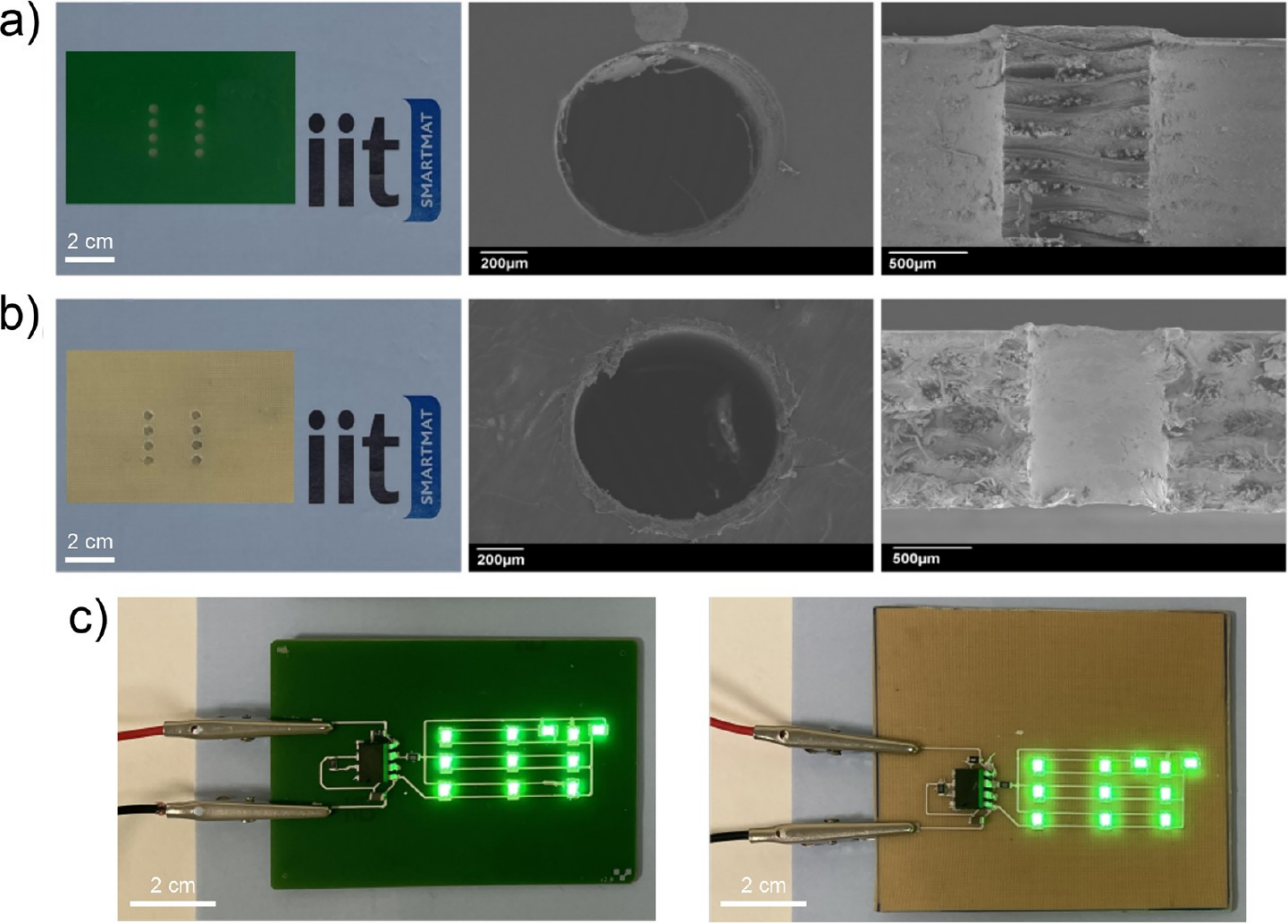
(a, b) Microdrilled holes, top and cross-section SEM views of a single hole, on FR4 and green PLA-CF substrates, respectively. (c) Circuit with dual-in-line and surface mount technology components. The circuit comprised a timer, LEDs, resistances, and capacitors. With both the FR4 and green PLA-CF substrate, the LEDs were lit up and blinking, applying a voltage of 4.5 V.

Drilling is the typical process made to PCBs to create mounting holes or vias to the substrate.^72^ It is fundamental to fix components and integrated circuits and create electrical connections between the different layers of the PCB. It is one of the most delicate and expensive steps of PCB fabrication. Thus, we verified if microdrilling can be realized efficiently on the green PLA-CF substrate and on the FR4 as a reference, as shown in Figure 4. Microscopically, the SEM top- and cross-sectional views of the obtained holes show identical size, equivalent resolution, and homogeneity on both surfaces. The formation of holes does not damage the substrate or create cracks along its surface and cross section, as shown in Figures 4a, 4b, and S10. The holes created on the PLA-CF substrate are shaped with a resolution similar to FR4.

As proof of principle for the function of the developed substrate for the green PCB, we designed a circuit with dual-in-line and surface mount technology components. The circuit was composed of a timer, LEDs, resistors, and capacitors. We drew identical circuits and attached the same components to the PLA-CF and FR4 substrates. On both PCBs, the circuit worked consistently, and the LEDs were lighting up (Figure 4c) and blinking with equivalent brightness, applying a voltage of 4.5 V, as shown in Video S2.

Finally, we tested the biodegradation in the soil of the developed PLA-CF substrate using FR4 and pure PLA as a reference (see Figure S11). This experiment was designed to assess the potential of our developed material in a scenario in which the PCB is accidentally discarded into the environment. For this preliminary investigation, we opted to examine the biodegradation of the substrate within a controlled laboratory setting, using soil as the testing medium. We set the experimental duration to 6 months, a substantial time frame in which we could observe the progression of biodegradation. The temperature and humidity were kept at 18 ± 2 °C and 60 ± 5%, respectively, during the experiment. The soil was periodically watered to keep the moisture at 60%. As expected, the commercial PCB and the pure biopolymer did not degrade inside the soil. PLA is fully compostable in an industrial compost plant, but it is not biodegradable at ambient conditions.^73^ Inserting cotton fabric into the matrix enabled the initiation of biodegradation in the soil at ambient conditions corresponding to a weight loss of 5% of the initial weight after 6 months of experiment, likely due to microorganisms’ digestion of cotton fabric, similar to what was previously reported in the literature for PLA composites with different vegetable waste.^74^ According to that publication, only the vegetable waste was degraded, while PLA kept the same molecular weight after 6 months buried in the soil.

## 3 Conclusions

The PCB industry is currently based on materials sourced from nonrenewable resources that have no end-of-life options other than landfilling, causing a significant environmental burden. In this regard, gradually turning to renewable raw materials and having different and more benign end-of-life options, such as composting, will reduce *e*-waste generation.

In this quest for sustainable PCBs, we realized a substrate for electronics layering of bio-based PLA and cotton fabric. The manufacturing consisted of compression molding, a potentially scalable solvent-free thermoforming technique already broadly employed in the polymer and composite sector. The PLA-CF green substrate’s main features were systematically compared with FR4, the most diffused commercially available PCB substrate, and are summarized in Table 1. The microtopography and cross-section structure of the PLA-CF are comparable with those of FR4. The interaction of the PLA-CF green substrate with water, ethanol, and acetone was similar to FR4; thus, conducting inks based on these solvents can be used for printing circuits. The related contact angles were measured in the range between ≈23° and 76°, preventing smearing and permitting the desired wetting of the conducting inks. Printed silver inks on top of the PCBs lead to precise inking, obtaining resistivities from 10^–6^ to 10^–7^ Ω·m. The developed green PCB was conformed/thermoformed to curved surfaces by heating it below 100 °C, close to the glass transition temperature of the biopolymer used, preserving the conductivity of the silver tracks. Microdrilling was performed successfully without damaging the bio-based substrates with undesired cracks. A proof-of-principle circuit with a dual-in-line integrated circuit timer and surface mount technology components such as LEDs, resistors, and capacitors were successfully assembled on top of the green PCB and demonstrated operationally.

**Table 1:** Comparison of the Salient Features of Standard Commercial Printed Circuit Board (FR4) with the Manufactured Substrate Made of PLA and Cotton Fabric (PLA-CF)^a^

property	FR4	PLA-CF
roughness (μm)	2.4	2.7
Young’s modulus (GPa)	≈20^76^	2
flexural modulus (GPa)	23	8
water contact angle (deg)	65	77
ethanol contact angle (deg)	21	27
acetone contact angle (deg)	11	23
*T*_g_ (°C)	130–180^70^	60^69^
resistivity (Ω·m)	8 × 10^–7^	11 × 10^–7^
*R*/*R*_0_ (after bending at 1.1 cm bending radius)	not measured	9
dielectric constant at 100 kHz	4.6	4.6
compostability	not compostable	compostable

a The glass transition temperatures (*T*_g_) refer to the pure polymeric matrices. Contact angles reported are measured 20 s after the liquid drop was released on the surface. The resistivity is measured on printed silver conductive lines on top of the substrates. The bending measurements were not performed on FR4 substrates since it was not possible to reshape such substrates with the same procedure used for the green PCB (i.e., at 80 °C).

Such proof-of-principle circuit demonstrates the applicability of the developed substrate, for instance, in energy-efficient hardware,^75^ which in combination with the possibility of conformable shaping can be suitable also for robotics systems requiring particular architectures. Low-power electronics are undoubtedly a highly popular and growing application field for the electronic industry and for low-power sensors for robots. In this context, systems do not typically require high thermal dissipation, mechanical shocks, and electrical currents, as circuits and systems are meant to be energy-efficient and battery-operated. These factors make our technology a tangible and ready-to-use solution for these applications. The main limitation for a broader application of the proposed technology may be the hygroscopic nature of the constituent material with which the green PCBs are made and possibly their low flame retardancy. The addition of flame-retardant and not hygroscopic additive to the PLA biopolymer may be a viable solution to reduce the importance of the above-mentioned drawbacks.

To achieve fully compostable electronics for mass production, further research is required to develop fully degradable inks and integrated components, which opens the way to huge research horizons. However, while the visionary development of fully compostable electronic components (e.g., microprocessors, passives, and sensors) can require decades of research investment; on the other hand, printed or additive manufactured tracks and glued components can be simply removed from the substrate using ad-hoc solvents which do not damage integrated electronic components. This feature could open the way for advanced design and reuse scenarios, especially for microprocessors and microcontrollers, the most complex and aggressively optimized integrated systems. After the removal of components and conductive tracks, the substrate could then undergo its composting process.

Lastly, the results presented in this work pose the basis for more sophisticated multilayer processes (practical to implement more complex circuits) that could be easily implemented in conjunction with the additive manufacturing process.

The development of compostable PCBs will require the implementation of appropriate *e*-waste management, but this would allow for the recovery of metals and semiconductors that could be further recycled, reducing the amount of *e*-waste in landfills.

## 4 Materials and Methods

### Materials

A commercially manufactured 1.6 mm thick FR4 substrate from Voltera was used as a reference. It was made of epoxy resin infused into glass fibers. Poly(lactic acid) (PLA) 2003D was purchased from NatureWorks. Plain-woven and bleached 100% cotton fabric, with 180 ± 5 g/m^2^ mass density, was bought form a local market and used for the experiments. The textile has 24 threads/cm density in the warp and weft direction. The Flex2 silver conductive ink was acquired from Voltera. According to the manufacturer, it was made of silver particles, diethylene glycol monoethyl ether acetate, and mineral spirits and had a viscosity of 5000–10000 cP at 25 °C. Milli-Q water was employed for the water contact angle measurements. Ethanol and acetone were purchased from Sigma-Aldrich.

### Methods

All of the tests were performed on at least three samples unless specified differently. Mean and standard deviations were then calculated.

#### Compression Molding

Materials were prepared in a 10 × 10 cm^2^ frame with a thickness of 1.6 mm. For pure PLA, 32 g of PLA pellets was molded between thin Teflon sheets at a temperature of 180 °C using a CARVER 4122 hydraulic press equipped with water cooling, heating for 5 min without applying pressure, and then 5 min under a load of 3 tons. For the PLA-CF composite, multilayer composite panels were produced using the stacking method, in which 15 g of PLA powder was spread evenly between five layers of cotton fabric, corresponding to a weight of 8 g. Then, it was molded between thin Teflon sheets at a temperature of 180 °C using a CARVER 4122 hydraulic press equipped with water cooling, heating for 5 min without applying pressure, and then 5 min under a pressure of 3 tons. The optimal number of layers was determined by considering the final volume of the compression molding mask and the density of the materials. The final composition made of six layers of PLA pellets alternating with five layers of cotton fabric permitted us to obtain a homogeneous cross section of the material without voids and optimally filled the volume of the mold during the production step, obtaining the best balance between the mechanical strength and structural integrity. The thickness of the mold was chosen to obtain about 1.6 mm, which is the same as that of the FR4 reference substrates.

#### Scanning Electron Microscopy (SEM)

The morphology of the materials was investigated by SEM in a JEOL JSM-6490LA microscope using the secondary electrons detector. The cross-sectional samples were immersed in liquid nitrogen and then fractured. The samples were attached to aluminum stubs by using carbon tape and were covered with 10 nm of gold by a sputter coating. The micrographs were acquired with 10 kV accelerating voltage and load current of 78 μA and at different magnifications.

#### Surface Profilometer

The roughness of substrates surface was acquired using a Zeta-20 optical profilometer (Zeta Instruments) working in confocal mode. The image size was 1920 × 1440 pixels, which with an objective of 20× corresponds to a field of view of 664 × 498 μm^2^. The *z* spacing in the vertical tomography used to reconstruct the 3D surface profile was set to match the *z* resolution of the 20× objective (500 nm).

#### Thermogravimetric Analysis

The thermogravimetric analysis was performed with a TGA Q500 instrument manufactured by TA Instruments. The measurements were taken with 3–5 mg of the samples placed in an aluminum pan and subjected to a flow of inert N_2_ gas at a rate of 50 mL/min. The pan was heated from 0 to 800 °C at a rate of 5 °C/min. The rate of weight loss was recorded as a function of time and temperature.

#### Differential Scanning Calorimetry

Differential scanning calorimetry thermograms were acquired with a DSC Q2500 (TA Instruments) from −30 to 240 °C under nitrogen flow (50 mL/min) at 20 °C/min by using nonhermetic aluminum pans.

#### Mechanical Properties

Tensile and bending properties were analyzed by means of uniaxial tensile tests in an INSTRON 3365 dynamometer equipped with a 2 kN load cell.

Before analysis, the samples were cut to a dog-bone shape. The dimensions in the straight region of the bone were 25.01 mm in length and 3.98 mm in width. After that, the thickness of each specimen was measured with a digital micrometer (Mitutoyo) with a 0.001 mm accuracy. The strain rate during the experiment was set at 5 mm min^–1^. At least five specimens of each sample were analyzed, and their Young’s modulus (MPa), tensile strength (MPa), and elongation at break (%) were obtained. Mean and standard deviations were calculated.

Three-point bending was performed on prismatic samples with a width *w* = 10 mm, setting a span *S* = 80 mm and loading rate of 5 mm min^–1^. From the stress–strain curves, the flexural modulus was extracted as the slope of the linear region and the flexural strength as the maximum value of the curve.

#### Contact Angle

The wetting properties of the substrates were investigated by the static water, ethanol, and acetone contact angle measurements, using a contact angle goniometer (OCAH-200, DataPhysics). First, a gastight 500 mL Hamilton precision syringe with a blunt needle of 0.52 mm internal diameter was used to deposit Milli-Q water, ethanol, and acetone droplets of 5 μL on the samples. Five droplets were deposited on different spots of each sample, and then the mean and standard deviation values were calculated.

#### Microdrilling

A Voltera V-One microdriller machine was utilized in the present study for performing microdrilling operations. Holes were made on each substrate with a maximum speed of 13000 rpm with a diameter of 0.7 mm, and the substrates were fixed with clamps of the driller machine. Two drilling hole columns were fabricated for this test on each substrate. The microdrilling was performed at room temperature under environmental conditions of 22 °C and 60% humidity. Tests were done with constant drill diameter and spindle speed.

#### Resistivity

First, silver ink was used to draw lines of about 2 cm long on each substrate. After printing, an annealing step of 30 min at 60 °C was performed to enhance the adhesion of the ink to the substrates. After this, the *I*–*V* curves were measured with a Keithley 2612A sourcemeter, and the resistances (*R*) were extracted. Then, the resistivity ρ was calculated using the geometries obtained from the SEM images and the formula^77^
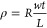
1where *R* is the resistance, *w* is the width, *t* is the thickness, and *L* is the distance between the probe tips. The resistance of the printed silver lines change was tested against decreasing bending radius.^78^ The PLA-CF substrate was gradually bent while heating the material at a temperature higher than the glass transition temperature (*T*_g_) of the PLA (at around 80 °C) and measured their conductivity in different bending states, i.e., at a bending radius of 1.8 1.1, and 0.7 cm. The setup used is visible in Figure S8.

For the scratch test, first, two lines of about 2 cm were drawn with silver ink on each substrate. The adhesion of silver conductive lines on PLA boards was evaluated by scratch tests on an Anton Paar Micro Combi scratch/indenter equipped with a Rockwell Sphero conical tip (radius *R* = 0.1 mm), and constant forces of 1, 2, and 3 N were applied, and the stage was moved transversally at a rate of 2 mm min^–1^. Images of the scratched lines were acquired on the Zeta-20 optical profilometer. The resistance before and after scratch was recorded with a Keithley 2612A sourcemeter.

#### Dielectric Constant

The dielectric test, also known as the “high potential” test, was performed to validate the effectiveness of the insulation capability of a component. First, four 1 cm^2^ plane capacitors were drawn with silver ink on each substrate. Next, using the Agilent 4263B LCR meter, a uniform voltage at different frequency ranges (0.1–100.0 kHz) was applied on the faces of the substrates using simple crocodile connectors. The equivalent capacitance *C* of each substrate was calculated at different frequencies, and then the dielectric value of each substrate was calculated using the formula
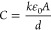
2where *d* is the distance between the faces, *A* is the area of the surface, *k* is the relative permittivity, and ε_0_ is the vacuum permittivity.

#### Biodegradation in Soil

The biodegradability of substrates and their respective controls was analyzed over a 6 month experiment following the methodology reported by Merino et al.^79^ Samples were cut into 4 cm^2^ square plates, put into a PE-mesh bag, and buried in the biodegradation media. For that a pot of 20 cm × 20 cm × 8 cm filled with soil for aromatic plants and vegetable garden (VIGORPLANT ITALIA S.R.L.) was used. The main physicochemical properties of the soil were a pH of 6.5, an electrical conductivity of 0.4 dS/m, a dry apparent density of 250 kg/m^3^, and a total porosity of 87% v/v. According to the supplier, it was composed of acidic sphagnum peat allowed in organic farming, green composted soil improver allowed in organic farming (produced from mixtures of composted and not chemically treated plant materials), and simple noncomposted vegetable soil improver allowed in organic farming, not chemically treated. The assay was conducted at 18 ± 2 °C and 60% ± 5% RH. The samples were initially dried for 24 h at 40 °C in a vacuum oven and weighed (*W*_0_). Then they were placed in handmade PE-mesh bags and buried in the soil. The pot with the samples was periodically watered along the time of the experiment in order to keep the soil moisture at 60%. The samples were removed at specific times: 1, 3, and 6 months. The soil attached to the samples was carefully removed with a brush, and samples were dried overnight in a vacuum oven at 40 °C and reweighted (*W_t_*). Finally, the weight loss (%) of each sample was determined via eq 3 and was represented as a function of time (months).^79^
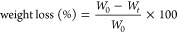
3Samples were analyzed in duplicate, and results were expressed as the average ± standard deviation.

## Supporting Information Available

Characterization results of DSC, TGA, SEM, profilometry, stress–strain and flexural resume table, contact angle resume table, *I*–*V* curve, bending setup, resume table dielectric constant, and biological oxygen demand (PDF)
Video S1: bending of the substrate with a heat gun (MP4)
Video S2: LEDs blinking on the printed circuit boards (MOV)

## Supplementary Material

el3c00799_si_002.mp4

el3c00799_si_003.mov
