# Supplementary material for: A Green Conformable Thermoformed Printed Circuit Board Sourced from Renewable Materials
Source: ACS Appl Electron Mater. 2023 Sep 18;5(9):5050–60. doi: 10.1021/acsaelm.3c00799 (PMC10537457; doi:10.1021/acsaelm.3c00799)
Supplement: Supplementary file 1 — el3c00799_si_001.pdf [file el3c00799_si_001.pdf]

Supporting information

A Green Conformable Thermoformed Printed Circuit Board Sourced from Renewable Materials

Amirsoheil Honarbari[£,§], Pietro Cataldi[£], Arkadiusz Zych[£], Danila Merino[£], Niloofar Paknezhad[£,#], Luca Ceseracciu[&], Giovanni Perotto[£], Marco Crepaldi[$], Athanassia Athanassiou[£]*.*

[£] Smart Materials, Istituto Italiano di Tecnologia, Via Morego 30, Genova, 16163, Italy.

[§] Dipartimento di Informatica, Bioingegneria, Robotica e Ingegneria dei Sistemi (DIBRIS), University of Genoa, Via all'Opera Pia 13, Genova, 16145, Italy

[#] Department of Biology, University of Rome "Tor Vergata", Via della Ricerca Scientifica, 00133 Rome, Italy

[&] Materials Characterization Facility, Istituto Italiano di Tecnologia, Genova, 16163, Italy

[$] Electronic Design Laboratory, Istituto Italiano di Tecnologia, Via Enrico Melen, 16152, Genova, Italy

Corresponding Author

*Pietro Cataldi: pietro.cataldi@iit.it; Athanassia Athanassiou: athanassia.athanassiou@iit.it

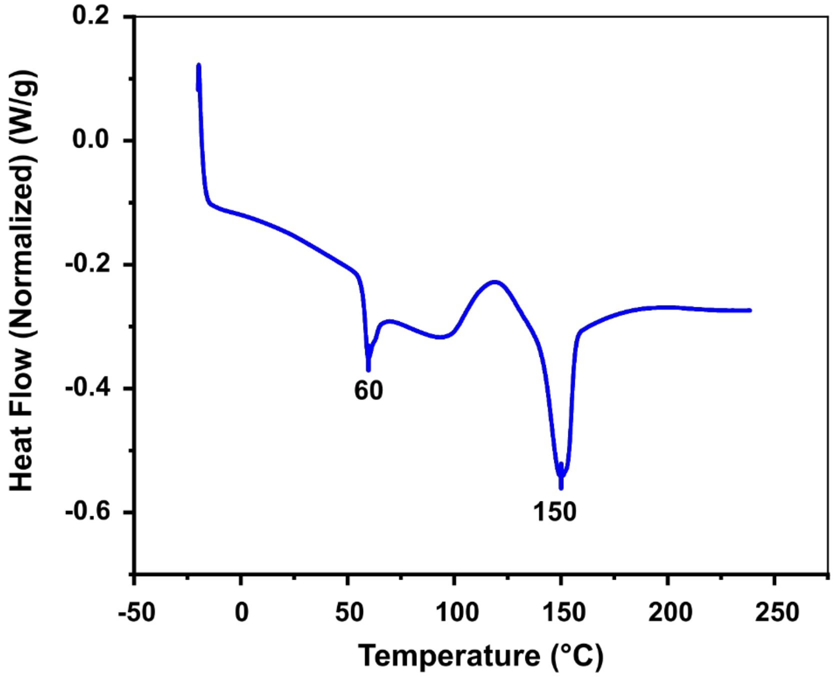

Figure S1. DSC analysis of PLA. At 60 °C the glass transition temperature of PLA is visible, while at 150°C the melting occurs.

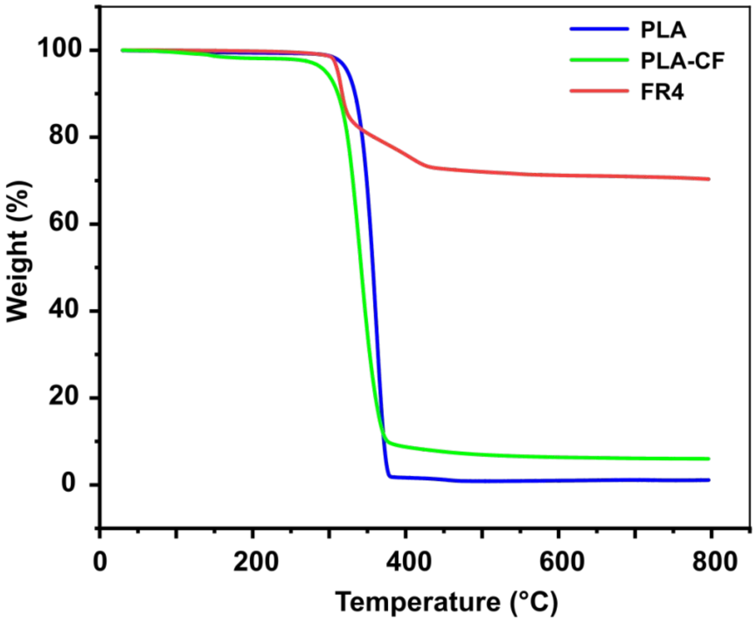

Figure S2. Thermogravimetric analysis of pure PLA, PLA-CF, and FR4 composites.

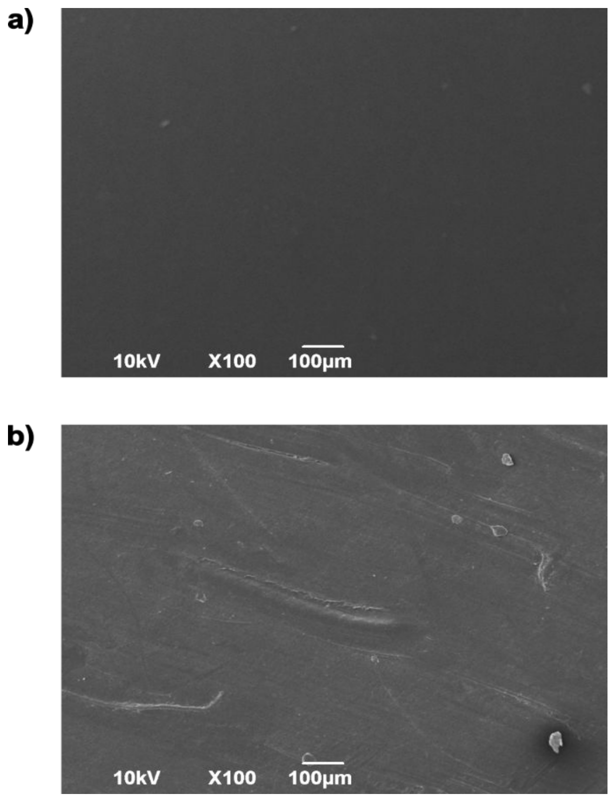

Figure S3. Top *SEM* view of the substrates, a) surface of FR4*;* b) surface of PLA-CF

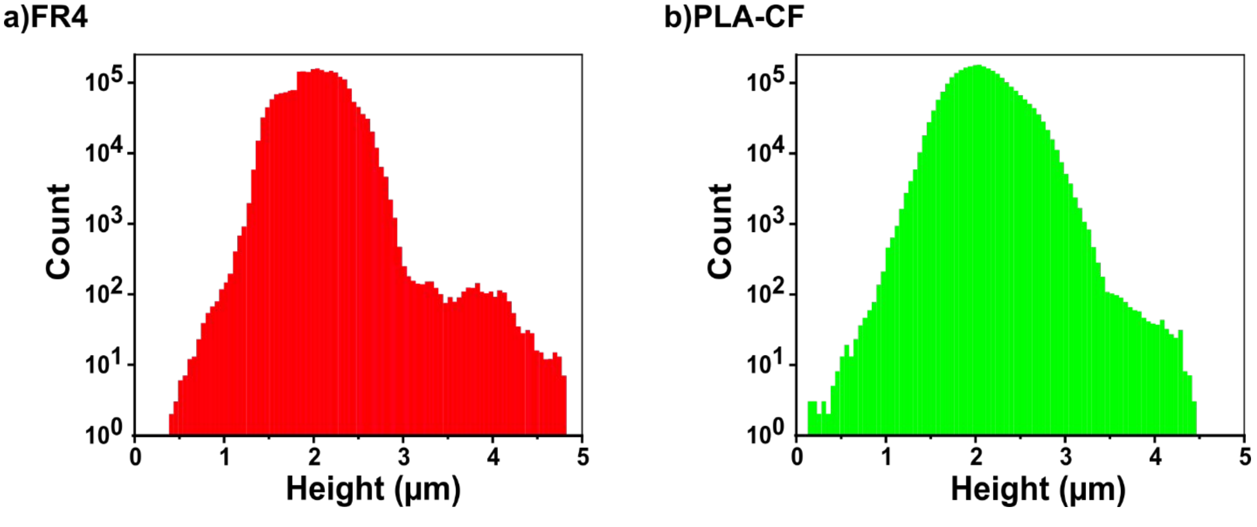

Figure S4. Profilometry of the surface, a) FR4 substrate. b) PLA-CF substrate. Both are extracted from the whole height profile area measured (see Figure 1b and 1c).

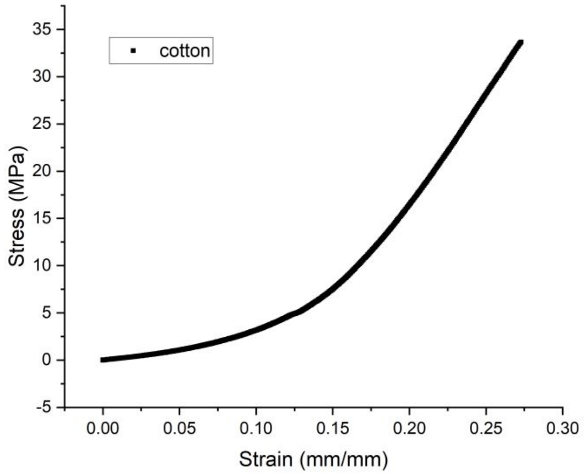

Figure S5. Stress strain curve of the pure cotton sample.

Table S1. resume of the tensile properties of the samples.

Sample	Young Modulus (MPa)	Stress at break (MPa)	Elongation at break (%)
PLA	1080 ± 43	54.4 ± 2.6	7.0 ± 0.2
PLA-CF	1827 ± 230	94.3 ± 5.6	11.2 ± 1.0

Table S2. resume of the flexural properties of the samples.

Sample	Flexural Modulus (MPa)
PLA	3072 ± 171
PLA-CF	7685 ± 1100
FR4	21732 ± 825

Table S3. contact angle of substrates with different solvents.

Water Contact angle				
Type of Substrate	Time (seconds)			
	5	10	20	30
FR4	69.08 ± 1.77°	67.01 ± 0.09°	64.51 ± 0.61°	64.15 ± 0.32°
PLA-CF	80.91± 0.73°	79.10± 0.51°	76.54 ± 1.28°	72.35 ± 0.95°
Ethanol Contact angle				
Type of	Time (seconds)			

Substrate	5s	10s	20s	30s
FR4	27.97 ± 4.09°	26.18 ± 4.01°	20.62 ± 3.69°	16.98 ± 4.4°
PLA-CF	32.99 ± 4.62°	27.43 ± 4.29°	27.20 ± 4.50°	26.43 ± 5.34°
Acetone Contact angle				
Type of Substrate	Time (seconds)			
	5s	10s	20s	
FR4	15.88 ± 2.94°	12.27 ± 3.20°	11.21 ± 3.46°	
PLA-CF	36.12 ± 5.90°	29.73 ± 4.59°	23.09 ± 4.59°	

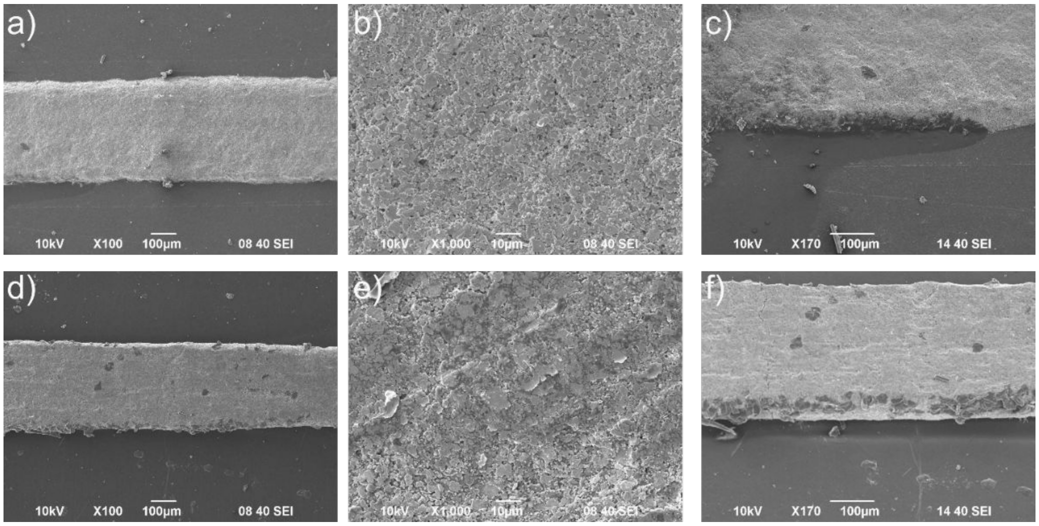

Figure S6. a) and b) SEM of the silver painted lines on top of the FR4. c) SEM of the thickness of the lines on FR4. d) and e) SEM of the silver painted lines on top of the green PCB. c) SEM of the thickness of the lines on the PLA-CF sample.

Table S4. Variation in R/R_0 due to the scratch performed at different loads on silver ink lines drawn on different substrates.

Type of substrates	R/R_0		
	1N	2N	3N
FR4	≈ 1	≈ 1	∞
PLA-CF	≈ 1	≈ 1	≈ 1

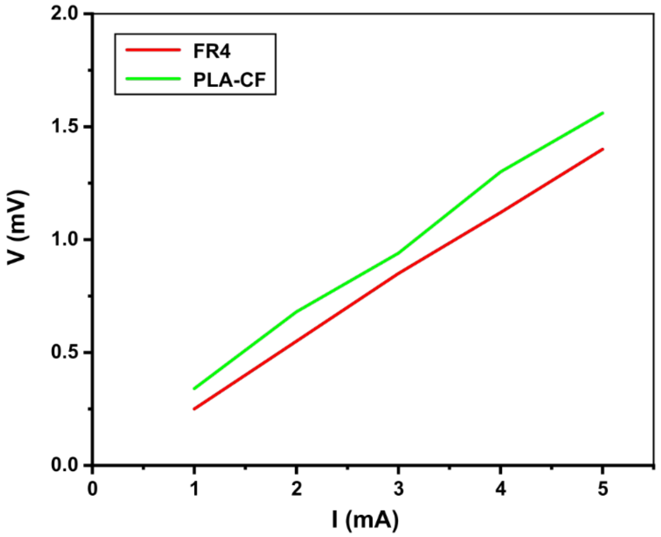

Figure S7. Example of the *I-V* graph of the silver ink printed on top of the substrates.

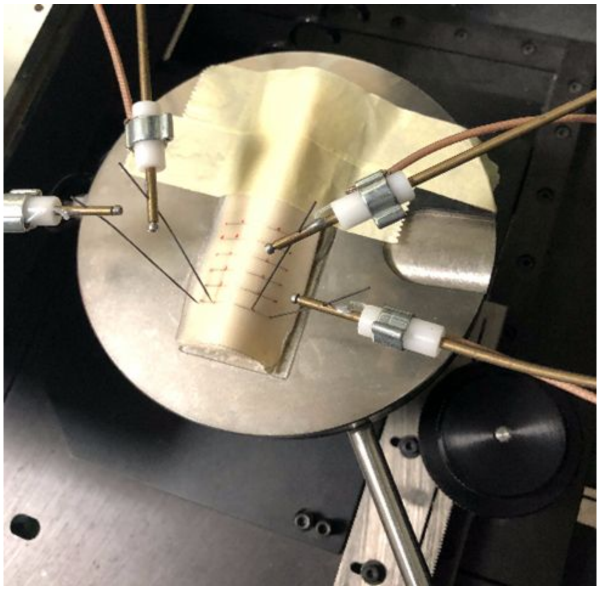

Figure S8. Setup used to test the variation of resistance of bent samples.

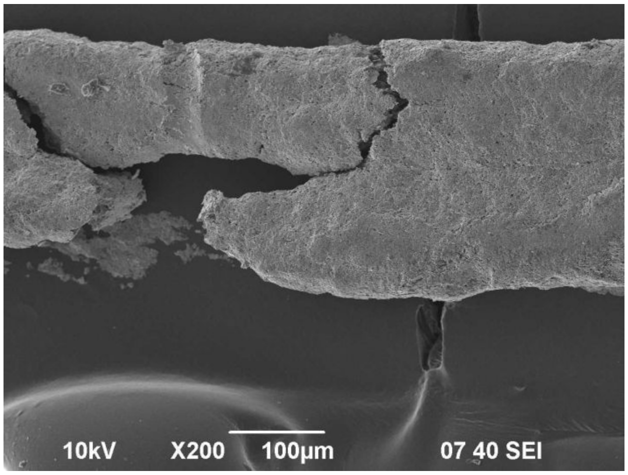

Figure S9. SEM of the silver track on top of the bent (i.e., 0.7 cm bending radius) substrate.

Table S5. Dielectric Constant of the green PCB and of the FR4 substrate at different frequencies.

Type of Substrates	Dielectric constant at 0.1kHz	Dielectric constant at 1kHz	Dielectric constant at 10kHz	Dielectric constant at 100kHz
PLA-CF	14.22 ± 1.14	8.30 ± 0.40	5.64 ± 0.32	4.60 ± 0.14
FR4	4.74 ± 0.05	4.70 ± 0.040	4.68 ± 0.04	4.63 ± 0.15

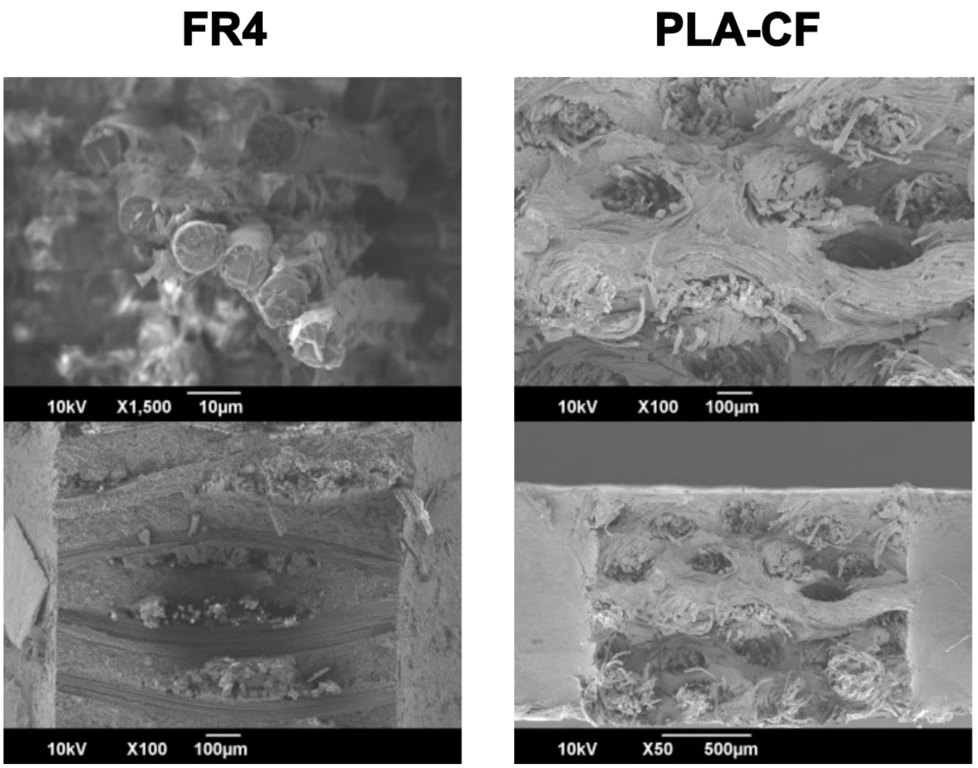

Figure S10. SEM of the holes obtained on the FR4 and PLA-CF.

Biodegradation in soil

This experiment was done over six months. The biodegradability of substrates and their respective controls was evaluated using the approach described by Merino et al[1]. This showed that the PLA-CF substrate could lose weight during this period, the cotton fabric inside the PLA-CF substrate has increased the biodegradation in soil of the samples in this experiment. but the FR4 substrate showed no signs of degradation or weight loss because it is made by epoxy resin and glass fiber for reinforcement.

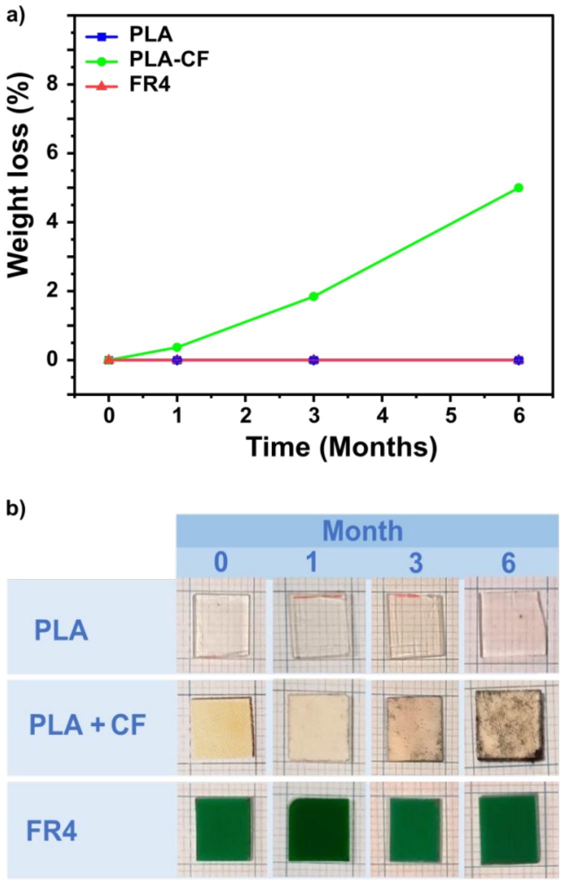

Figure S11. a) Weight loss in soil of the substrates versus time of contact with soil. b) Photos of the substrates at different time point in soil.

Supporting information references:

[1] D. Merino, A. Y. Mansilla, C. A. Casalongué, V. A. Alvarez, Journal of Polymers and the Environment 2019, 27, 1959.
